# Theranostic Tripartite Cancer Terminator Virus for Cancer Therapy and Imaging

**DOI:** 10.3390/cancers13040857

**Published:** 2021-02-18

**Authors:** Praveen Bhoopathi, Anjan K. Pradhan, Santanu Maji, Swadesh K. Das, Luni Emdad, Paul B. Fisher

**Affiliations:** 1 Department of Human and Molecular Genetics, School of Medicine, Virginia Commonwealth University, Richmond, VA 23298, USA; anjan.pradhan@vcuhealth.org (A.K.P.); santanu.maji@vcuhealth.org (S.M.); swadesh.das@vcuhealth.org (S.K.D.); luni.emdad@vcuhealth.org (L.E.); 2 VCU Institute of Molecular Medicine, School of Medicine, Virginia Commonwealth University, Richmond, VA 23298, USA; 3 VCU Massey Cancer Center, School of Medicine, Virginia Commonwealth University, Richmond, VA 23298, USA

**Keywords:** tripartite, cancer terminator virus, MDA-7/IL-24, theronostic, imaging, cancer cell death, prostate cancer, breast cancer

## Abstract

**Simple Summary:**

An optimum cancer therapeutic virus should embody unique properties, including an ability to: Selectively procreate and kill tumor but not normal cells; produce a secreted therapeutic molecule (with broad-acting anti-cancer effects on primary and distant metastatic cells because of potent “bystander” activity); and monitor therapy non-invasively by imaging primary and distant metastatic cancers. We previously created a broad-spectrum, cancer-selective and replication competent therapeutic adenovirus that embodies two of these properties, i.e., specifically reproduces in cancer cells and produces a therapeutic cytokine, MDA-7/IL-24, a “cancer terminator virus” (*CTV*). We now expand on this concept and demonstrate the feasibility of producing a tripartite *CTV* (*TCTV*) selectively expressing three genes from three distinct promoters that replicate in the cancer cells while producing MDA-7/IL-24 and an imaging gene (i.e., luciferase). This novel first-in-class tripartite “theranostic” *TCTV* expands the utility of therapeutic viruses to non-invasively image and selectively destroy primary tumors and metastases.

**Abstract:**

Combining cancer-selective viral replication and simultaneous production of a therapeutic cytokine, with potent “bystander” anti-tumor activity, are hallmarks of the cancer terminator virus (*CTV*). To expand on these attributes, we designed a next generation *CTV* that additionally enables simultaneous non-invasive imaging of tumors targeted for eradication. A unique tripartite *CTV* “theranostic” adenovirus (*TCTV*) has now been created that employs three distinct promoters to target virus replication, cytokine production and imaging capabilities uniquely in cancer cells. Conditional replication of the *TCTV* is regulated by a cancer-selective (truncated *PEG-3*) promoter, the therapeutic component, MDA-7/IL-24, is under a ubiquitous (*CMV*) promoter, and finally the imaging capabilities are synchronized through another cancer selective (truncated *tCCN1*) promoter. Using in vitro studies and clinically relevant in vivo models of breast and prostate cancer, we demonstrate that incorporating a reporter gene for imaging does not compromise the exceptional therapeutic efficacy of our previously reported bipartite *CTV*. This *TCTV* permits targeted treatment of tumors while monitoring tumor regression, with potential to simultaneously detect metastasis due to the cancer-selective activity of reporter gene expression. This “theranostic” virus provides a new genetic tool for distinguishing and treating localized and metastatic cancers.

## 1. Introduction

Noninvasive imaging for cancer diagnosis, as a means of guiding therapy, is critical for improving clinical outcomes in patients with diverse localized and distant (metastatic) tumors [1,2,3]. Although there have been significant refinements in imaging approaches and therapies for cancers, detection and therapy for metastasis remain imprecise with limited effectiveness, [4]. Recent strategies are successfully combining both therapy and diagnosis in the same therapeutic vector/molecule, an approach termed “theranostics.” Using viral vectors that are specific for cancer (by using a cancer-specific promoter to control critical regulators of viral replication) and that also produce therapeutic agents with expanded field effects, i.e., “bystander” antitumor activity, and simultaneously promote immune modulation, inhibit angiogenesis, and synergize with currently used therapeutics would in principle provide exceptional opportunities to develop “theranostics” for both treating and detecting (by using a second cancer-selective promoter to drive expression of a luciferase reporter gene) primary and metastatic cancers [4].

Multiple studies accentuate the value of immunotherapy in the treatment of specific patients with advanced cancers [5,6]. Melanoma differentiation-associated gene-7/Interleukin-24 (*mda-7/IL-24*) is an exceptional therapeutic cytokine member of the IL-10 gene family [7,8], with immune-modulating activity [9,10,11]. *mda-7/IL-24* promotes both direct antitumor activity by inducing cancer-specific apoptosis as well as indirect antitumor activity by provoking anti-angiogenesis activity [12,13,14,15], in vitro and in vivo in preclinical animal models and in a Phase I clinical trial in patients with advanced cancers (includes breast carcinoma, colon carcinoma, squamous cell carcinoma of the head and neck, melanoma, large cell lymphoma, adrenal carcinoma, transitional cell carcinoma). [15,16,17,18,19,20]. Another attribute of MDA-7/IL-24 is its ability to synergize with radiation, chemotherapy, antibody and/or immune therapy approaches resulting in enhanced eradication of cancer cells [7,8,13,15,21,22,23,24,25,26,27,28,29]_._ MDA-7/IL-24 selectively induces apoptosis and/or toxic autophagy in a broad spectrum of cancer cell types, including breast cancer [27,29,30,31,32,33,34], prostate cancer [25,35,36,37,38], melanoma [31,34,39,40,41,42,43,44,45], glioblastoma multiforme [22,24,46,47,48,49], neuroblastoma [50], lung [14,21,23,26,28], and numerous other cancers [7,8,13,15,17]. In contrast, it does not promote similar changes in normal cells and has not shown toxicity in pre-clinical animal models of cancer [7,8,13,15,17] or in patients with advanced cancers that received direct intratumoral injection with an *mda-7/IL-24* expressing replication incompetent type 5 adenovirus in a Phase I clinical trial [15,17,18,19,20].

MDA-7/IL-24 promotes cancer-selective apoptosis by inducing endoplasmic reticulum (ER) stress, which causes up-regulation of BiP/GRP78, induction of pro-apoptotic proteins (such as BAX), and repression of anti-apoptotic proteins (such as BCL-2) [51,52,53,54]. Another mechanism by which MDA-7/IL-24 promotes cell death is through toxic autophagy, which is associated with the induction of Beclin-1 and the cleavage of ATG5 [31,38]. A key element in MDA-7/IL-24’s effectiveness as an anticancer agent is its ability to be secreted by both normal and cancer cells resulting distinctively in the killing of primary and distant (metastatic) cancer cells [7,8,13,15,44,52,53,55,56]. The molecular/biochemical basis for this profound “bystander” anticancer effect is partly through secreted MDA-7/IL-24 protein binding to dimeric IL-20/IL-22 surface receptors inducing production of MDA-7/IL-24 protein via a paracrine-autocrine feedback loop in adjacent and distant tumor cells [52,57]. 

To improve the delivery and therapeutic efficacy of MDA-7/IL-24 we created bipartite conditionally replicating adenoviruses expressing *mda-7/IL-24,* also known as a cancer terminator virus (*CTV*s) [44,58,59,60]. In these adenoviruses, E1A and E1B expression is controlled by a cancer-selective genetic element, either a truncated progression elevated gene-3 (*PEG-3*) promoter [61,62,63] or a truncated cellular communication network factor 1 (*tCCN1*) promoter [64]. The *CTV* induces cancer-selective replication and a second *CMV* promoter promotes *mda-7/IL-24* expression solely in cancer cells as a function of virus replication [44,58,59,60]. This results in profound anticancer activity, which is greater than the observed following infection with a non-replication adenovirus expressing *mda-7/IL-24*, in vitro in established cancer cell lines, primary cancer-derived tumor cells, and patient-derived human PDX tumor cells [44,45,58,59,60]. Of added relevance supporting the potential of *mda-7/IL-24* and the *CTV* for use in cancer gene therapy are the observations that the *CTV* actively suppresses growth and kills cancer cells in vivo in human tumor xenografts in nude mice, murine syngeneic tumors in immune competent mice and genetically engineered transgenic mice [44,45,58,59,60]. These broad-ranging cancer-killing effects are observed in the absence of toxicity in pre-clinical animal models and in patients with advanced cancers in a Phase I trial [17,18,19,20]. 

We now describe a next generation *CTV,* a tripartite *CTV* (*TCTV*), in which replication is controlled by the truncated *PEG-3* promoter, expression of *mda-7/IL-24* is under transcriptional regulation of the *CMV* promoter, and a luciferase gene is regulated by a truncated *tCCN1* promoter. The *TCTV* displays equivalent gene expression, biological activity (growth-suppression and apoptosis-induction) in vitro and in vivo as the *CTV*, with additional expression of luciferase only by the *TCTV.* In vivo studies confirm imaging and therapeutic competences in both nude mice and syngeneic transgenic animals containing xenotransplant tumor cells or spontaneously developing breast cancer. Additionally, the *TCTV*, as well as the *CTV*, do not express any of the genes or display any negative biological effects in normal primary breast mammary epithelial cells or immortal prostate epithelial cells. Our results confirm that the addition of a reporter gene to a conditionally replicating *CTV* does not compromise therapeutic efficacy. These observations support the ability to develop a “theranostic” tripartite therapeutic cancer terminator virus that retains cancer-selective replication, targeted therapeutic cytokine expression and non-invasive imaging potential.

## 2. Results

### 2.1. Construction of a Tripartite Cancer Terminator Virus (Ad.5-TCTV)

**Figure 1:**
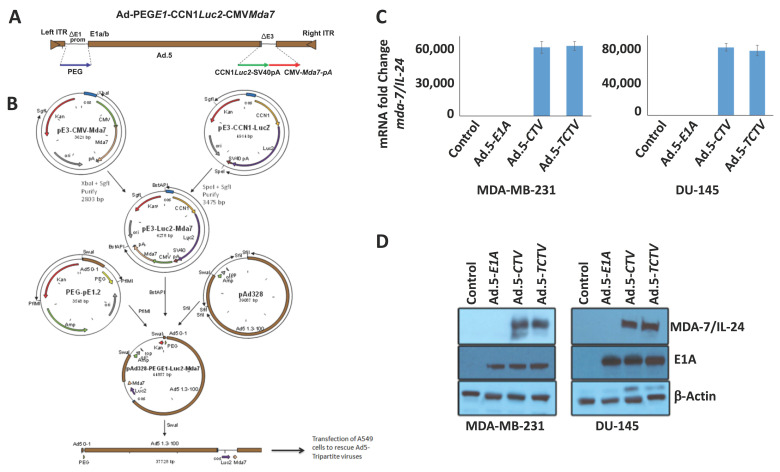
Construction and characterization of Ad.5-*TCTV*. (**A**) Schematic diagram representing the gene sequence in the vector used to generate the *TCTV*. A truncated *PEG*-Prom drives type 5 adenovirus replication and a truncated *tCCN1*-Prom drives luciferase expression. (**B**) Schematic representation of the cloning procedure used to construct the Ad.5-*TCTV*. (**C**) MDA-MB-231 and DU-145 cells were infected with either Ad.5-*E1A* (25 pfu), Ad.5-*CTV* (25 pfu), or Ad.5-*TCTV* (25 pfu) for 48 h. Total RNA was extracted using Trizol reagent, and reverse transcriptase PCR was performed for assessment of *mda-7/IL-24* mRNA transcript levels. *GAPDH* served as a loading control. (**D**) Western blotting for E1A and MDA-7/IL-24 protein expression was done using cell lysates with specific antibodies.

We previously engineered a conditionally replication-competent bipartite type 5 adenovirus, in which the cancer-selective truncated *PEG-3* promoter controlled the viral replication and the cytomegalovirus (*CMV*) promoter regulated the *mda-7/IL-24* gene expression (Ad.5-*PEG-E1A-CMV-MDA-7*), a cancer terminator virus (*CTV*) [44,58,59,60]. We documented further that this Ad.5-*CTV* displayed robust expression in a broad spectrum of different cancer cells and exhibited profound anti-tumor activities in a wide variety of pre-clinical cancer models, with negligible expression and activity in normal cells [44,58,59,60,65]. These observations prompted us to determine if we could develop a next generation tripartite “theranostic” virus with regulated expression of three genes under control of three distinct promoters in a cancer-selective manner (outline of construction in Figure 1A). The engineering of such a virus (Ad.5-*PEG-E1A-tCCN1-Luc-CMV-mda-7*), with a truncated *PEG*-Prom-driving E1A, a truncated *tCCN1*-Prom-regulating luciferase production, and a *CMV*-Prom-driving *mda-7/IL-24*, was accomplished in successive steps (Figure 1B). Details of this construction are provided in Materials and Methods. It should be noted that attempts to produce a simpler tripartite “theranostic” virus in which the truncated *PEG*-Prom was used to control both viral replication and luciferase production was unsuccessful due to potential recombination between the *PEG-3* sequences thereby preventing virus development.

The *TCTV* construct was used to generate Ad.5-*TCTV* and evaluated for its ability to produce *mda-7/IL-24* mRNA and E1A and MDA-7/IL-24 proteins following infection of human cancer cells (Figure 1C,D). Human breast (MDA-MB-231) and prostate (DU-145) cancer cells were infected with Ad.5-*E1A* (an adenovirus expressing E1A driven by the truncated PEG-3-Prom), Ad.5-*CTV,* or Ad.5-*TCTV* and mRNA and protein expression was determined 48 h later. Under these experimental conditions, Ad.5-*E1A* expressed only the Ad5 E1A protein, whereas Ad.5-*CTV* and Ad.5-*TCTV* infection resulted in comparable levels of expression of Ad5 E1A (protein) and MDA-7/IL-24 (mRNA and protein) (Figure 1C,D). These results confirmed that creating a tripartite Ad.5, i.e., Ad.5-*TCTV*, did not compromise its ability to produce *Ad5 E1A* or the *mda-7/IL-24* transgene following the infection of cancer cells, which were expressed at comparable levels as observed following infection with a bipartite Ad.5-*CTV* (Figure S1).

### 2.2. Ad.5-CTV and Ad.5-TCTV Inhibit Cancer Cell Proliferation without Affecting Normal Cells

**Figure 2:**
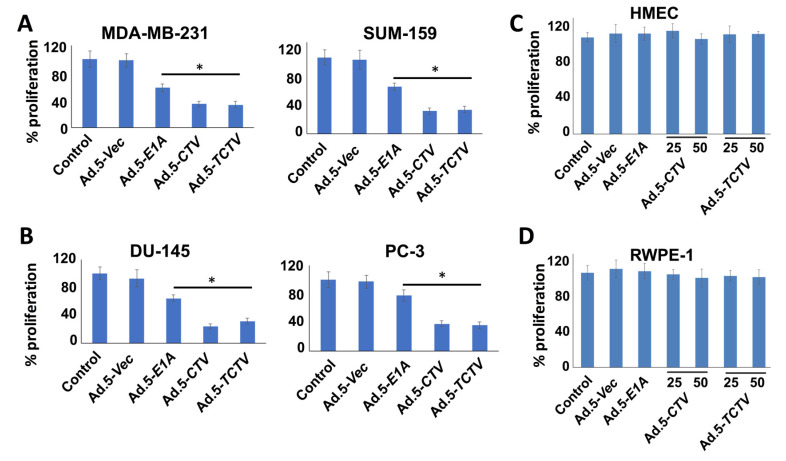
Ad.5-*TCTV* inhibits cancer cell proliferation to a similar extent as Ad.5-*CTV*. (**A**–**D**) The indicated cell types (5000 cells), (**A**) human breast cancer (MDA-MB-231 and SUM-159); (**B**) human prostate cancer (DU-145 and PC-3); (**C**) primary normal human breast epithelial (HMEC); and (**D**) normal immortal prostate epithelial (RWPE-1) cells were plated in 96-well plates and infected with either Ad.5-*E1A* (25 pfu), Ad.5-*CTV* (25 pfu), or Ad.5-*TCTV* (25 pfu) for 72 h. These cells were then treated with 20 μL of MTT in phosphate-buffered saline (PBS) and incubated further for 4 h. Media was then removed and formazan crystals were dissolved using dimethyl sulphoxide (DMSO) (100 μL). A microplate reader was used to measure the absorbance at 550 nm and the results are presented as percent proliferation with the comparison of cells treated with vehicle. Points, mean of triplicate experiments; bars, S.E. * *p* < 0.05 vs. control.

We evaluated next the effects of Ad.5-*CTV* and Ad.5-*TCTV* on cancer cell growth using MTT proliferation assays. Breast and prostate cancer cells were infected with similar active viral particles (plaque forming units; pfu) of Ad.5-*E1A*, Ad.5-*CTV,* or Ad.5-*TCTV* for 72 h and MTT assays were performed. Although Ad.5-*E1A* inhibited cell proliferation to some extent through viral replication (oncolytic activity), the combination of viral replication and expression of *mda-7/IL-24* using the Ad.5-*CTV* or Ad.5-*TCTV* resulted in an enhanced decrease in cell proliferation as compared with Ad.5-*E1A* (Figure 2A,B). Similar infection with any of these viruses did not inhibit proliferation in primary breast epithelial cells or normal immortal prostate epithelial cells (Figure 2C,D). These findings indicate that, as observed previously with Ad.5-*CTV* [44,58,59,60], in Ad.5-*TCTV*-infected cells the truncated *PEG*-promoter allowed adenovirus replication specifically in cancer cells, whereas normal cells were protected from growth inhibition because of the absence of adenovirus replication (due to reduced levels of AP1 and PEA3 transcription factors) [55,62], and tumor suppressor (*mda-7/IL-24*) transgene expression. Most importantly, adding a third component (an imaging cassette) to the *CTV* driven by the *tCCN1* promoter (*TCTV*) did not disrupt the cancer-selective growth inhibitory activity of this tripartite virus.

### 2.3. Ad.5-TCTV and Ad.5-CTV Infection Induce Comparable Levels of Cell Death in Cancer Cells 

**Figure 3:**
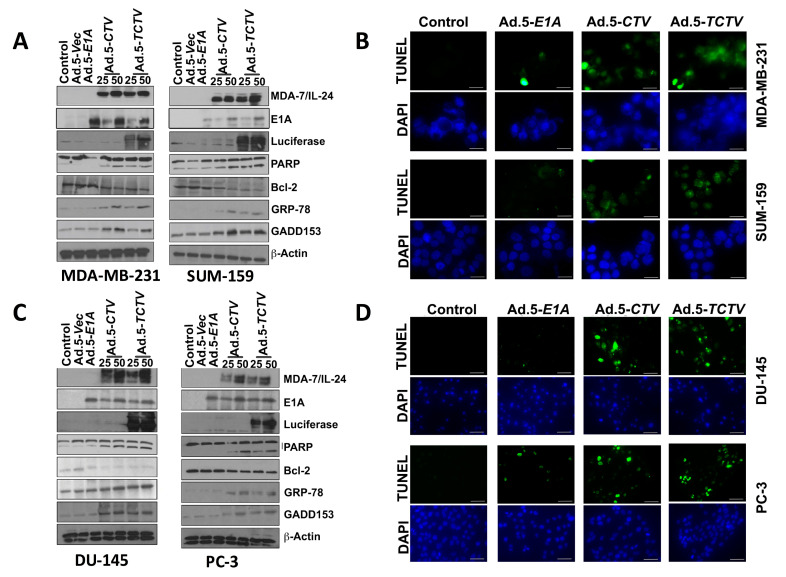
Ad.5-*TCTV* induces ER stress and apoptosis in cancer cells. (**A**) Breast cancer cells (MDA-MB-231 and SUM-159) treated with 25 pfu of Ad.5-*E1A* or the indicated pfu of Ad.5-*CTV* or Ad.5-*TCTV* for 72 h. Cells were then collected and Western blotting analysis was performed for signaling molecules using specific antibodies and β-actin served as a loading control. (**B**) Breast cancer cells were cultured in 8-well chamber slides and infected with 25 pfu of Ad.5-*E1A*, Ad.5-*CTV,* or Ad.5-*TCTV* for 72 h. Cells were fixed and TUNEL assays were performed. Data presented as TUNEL positive cells in a defined microscopic field as compared with un-treated control cells. (**C**) Prostate cancer cells (DU-145 and PC-3) were treated as described in **A**, cells were then collected and Western blotting analysis was performed for signaling molecules using specific antibodies and β-actin served as a loading control. (**D**) Prostate cancer cells were treated as described in **B** and cells were fixed and TUNEL assays were performed. Data presented as TUNEL positive cells in a defined microscopic field as compared with un-treated control cells. Scale bar: 200 μM.

**Figure 4:**
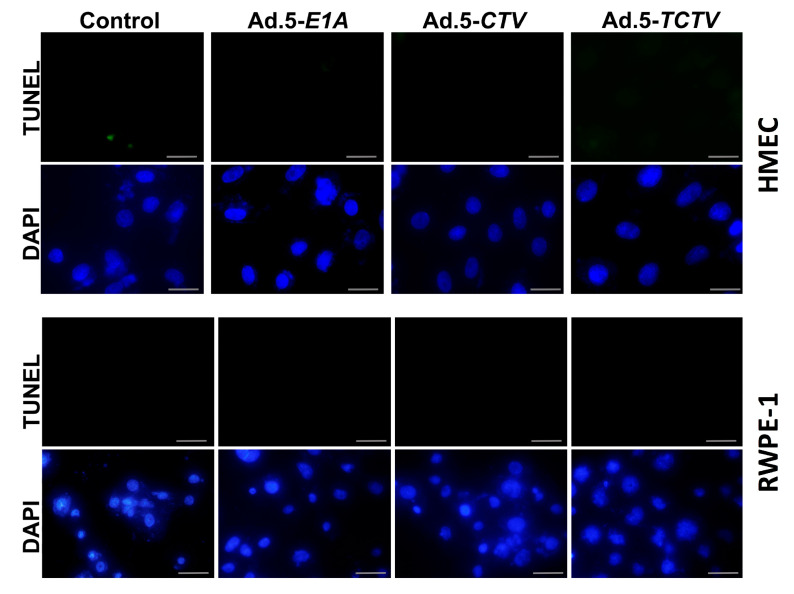
The *TCTV* does not induce apoptosis in normal breast and prostate epithelial cells. Normal primary breast epithelial (HMEC) and normal immortal prostate epithelial (RWPE-1) cells were cultured in 8-well chamber slides and treated with 25 pfu of Ad.5-*E1A*, Ad.5-*CTV,* or Ad.5-*TCTV* for 72 h. Cells were fixed and TUNEL assays were performed. Data presented as TUNEL positive cells in a defined microscopic field as compared with the un-treated control cells. Scale bar: 200 μM.

Previous studies confirmed that ectopic expression of *mda-7/IL-24* induces endoplasmic reticulum (ER) stress and apoptosis in a wide array of cancer cells through modulation of relevant signaling pathway molecules [7,8,13,15,56]. Accordingly, we determined whether Ad.5-*TCTV* infection of cancer cells instigated similar molecular pathway alterations as previously observed when *mda-7/IL-24* was delivered via an Ad.5-*CTV*. As shown in Figure 3A,C, infection with either Ad.5-*CTV* or Ad.5-*TCTV* at 25 or 50 pfu increased the MDA-7/IL-24 transgene protein expression. In many cancers, *mda-7/IL-24* enhances pro-apoptotic and decreases anti-apoptotic Bcl-2 family member protein expression and elevates ER stress-related protein expression [8,25,27,36,51,52,53,54,66,67]. Infection with Ad.5-*CTV* or Ad.5-*TCTV* caused comparable changes in pro- and anti-apoptotic proteins, i.e., decreased BCL-2 and increased PARP expression, in breast and prostate cancer cells, (Figure 3A,C). Additionally, similar increases in the ER-stress-related markers, GRP-78 and GADD153, were evident following Ad.5-*CTV* or Ad.5-*TCTV* infection (Figure 3A,C). Densitometry analyses of Western blots also showed that there are no major changes in the pattern of protein expression in Ad.5-*CTV* and Ad.5-*TCTV* treated cell lines (Figure S2A–D). Apoptosis induction by Ad.5-*CTV* or Ad.5-*TCTV* in cancer cells was also monitored by TUNEL assays, where a comparable increase in TUNEL-positive cells was evident following the infection with both CTVs as compared to mock- or Ad.5-*E1A*-infected cells (Figure 3B,D). In contrast, infection of normal breast or prostate epithelial cells with Ad.5-*CTV* or Ad.5-*TCTV* did not result in TUNEL positivity (Figure 4). These results support adenovirus-restricted replication and toxicity in cancer cells, while sparing normal cells. Additionally, luciferase (imaging component of the *TCTV*) expression was only evident in Ad.5-*TCTV*-infected cancer cells (Figure 3A,C). Taken together, the retention of cancer-cell-specific growth suppression and induction of apoptotic markers by the Ad.5-*TCTV* with simultaneous expression of luciferase confirms that addition of an imaging component (luciferase gene regulated by the *tCNN1* promoter) in the tripartite adenovirus does not hamper the functionality of this tripartite *CTV*.

### 2.4. Ad.5-TCTV Inhibits Tumor Growth while Permitting Tumor Imaging 

A key element of this study was demonstrating that the Ad.5-*TCTV* retained both tumor inhibitory properties and imaging properties in vivo in pre-clinical animal tumor models. To achieve these objectives, we focused on tumor xenograft models in nude mice and a transgenic mouse breast cancer model, representing primary tumor formation and potential tumor metastasis. *mda-7/IL-24* has well-established tumor-suppressor and apoptosis-promoting properties in a broad spectrum of human cancer cells [7,8,13,15]. The molecular mechanism by which *mda-7/IL-24* induces apoptosis is diverse involving different pathways in specific tumor contexts [8,13,15,56]. The “theranostic” properties of Ad.5-*TCTV* in vivo were examined in nude mice containing established human MDA-MB-231 breast cancer xenografts or established Hi-Myc mouse prostate tumor-derived cancer cells (Hi-Myc mu-PDX) established on both upper and lower animal flanks. Additionally, therapeutic activity of the Ad.5-*TCTV* was evaluated in the MMTV-PyMT (Mouse mammary tumor virus polyoma middle T) transgenic mouse model, which develops tumors in multiple mammary glands. 

**Figure 5:**
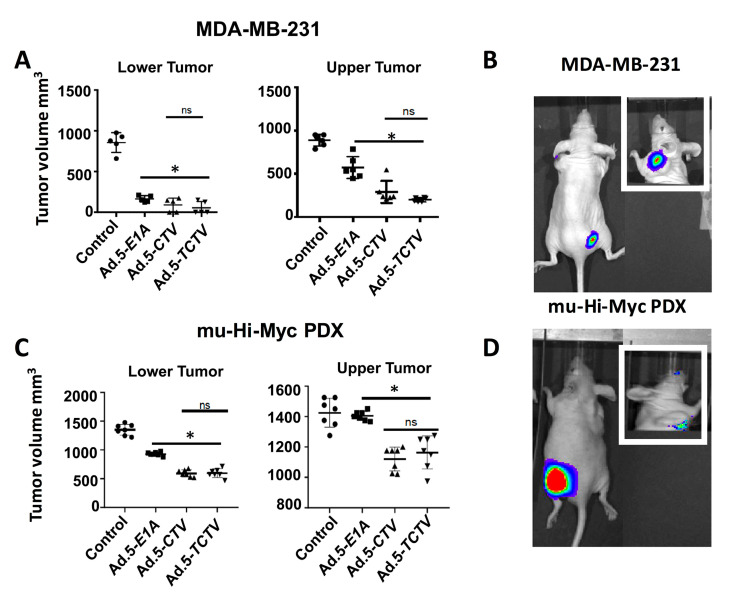
Ad.5-*TCTV* inhibits tumor growth to a comparable level of Ad.5-*CTV* and additionally permits non-invasive imaging of tumor growth *in vivo*. MDA-MB-231 (breast) and mu-Hi-Myc PDX (prostate) cancer cells were implanted subcutaneously in the lower and upper flanks of nude mice and lower tumors were treated with eight intratumoral injections of solvent (mock treatment-Control), Ad.5-*E1A*, Ad.5-*CTV,* or Ad.5-*TCTV*. A total of five animals were studied in each group. Once the tumors in untreated mice reached maximum IACUC acceptable limits, mice were euthanized and tumors were collected, fixed in formalin, and embedded in paraffin. (**A** and **C**) Tumor volumes from the lower (injected tumors) and upper flanks (un-injected tumors) were measured and the results are presented in a graphical manner. The line represents average of all tumor volumes of the group. * *p* < 0.05 vs. control, ns: no significant difference between Ad.5-*CTV* or Ad.5-*TCTV* group. (**B** and **D**) Ad.5-*TCTV* injected tumors were visualized using BLI (IVIS imager). Inset, BLI image of un-injected tumors following injection of lower flank tumor with Ad.5-*TCTV.*

**Figure 6:**
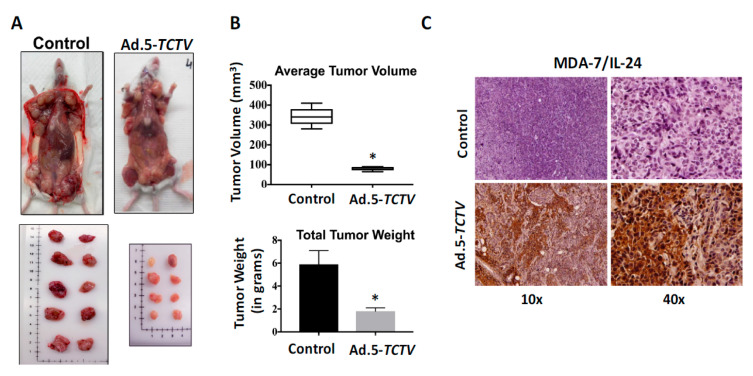
Ad.5-*TCTV* inhibits tumor growth in the MMTV-PyMT model. Mice treated intratumorally with Ad.5-*CTV* as described in methods or untreated controls were monitored for tumor burden over 4-weeks following first appearance of tumors. (**A**) Shown are representative photographs of tumors present in MMTV-PyMT mice untreated or treated with Ad.5-*TCTV* at the time of euthanasia. In each of the images, image dimensions are adjusted roughly to match the scale. (**B**) Tumor volumes and weights were quantified and the average tumor volume and total tumor weight are presented in a graphical manner. * *p* < 0.05 vs. control. (**C**) Immunohistochemical analysis of MDA-7/IL-24 in control and Ad.5-*TCTV* treated tumor sections.

For the MDA-MB-231 and Hi-Myc mu-PDX xenograft studies, 2 × 10^6^ cells were injected in the upper and lower flank of 4- to 6-week old athymic nude mice. Experiments began when palpable tumors of ~75 mm^3^ were detected in the lower and upper flanks of animals. The lower flank tumors were mock treated or infected with 1 × 10^8^ vp (a total of 8 intratumoral injections) of Ad.5-*E1A*, Ad.5-*CTV,* or Ad.5-*TCTV*, three times per week for the first two weeks, and two times in the third week. No injections were given to the upper-flank tumors. Once the mock-treated tumors reached maximum allowable size experiments were terminated. Injections of Ad.5-*E1A*, Ad.5-*CTV,* or Ad.5-*TCTV* resulted in inhibition of tumor growth after three injections and with eight injections some of the injected tumors were no longer evident (Figure 5A,C). Although Ad.5-*E1A* inhibited the growth of tumors on the lower flank (injected tumor), it had a negligible inhibitory effect on the tumors on the upper flank, which was not statistically significant. In contrast, Ad.5-*CTV* and Ad.5-*TCTV* significantly inhibited tumor growth on both flanks, highlighting the significant “bystander” anti-tumor function of *mda-7/IL-24* as well as possible virus and/or *mda-7/IL-24* transfer from the infected to the non-infected tumor site (which was anticipated based on the compromised immune system in these nude mice) (Figure 5). The observation that intratumoral injection of *CTV* and *TCTV* dramatically reduced the primary tumor and noticeably inhibited the distant tumor suggests that this approach may prove applicable for positively treating aggressive cancers with distant metastases. As predicted, we were able to image the tumors injected with Ad.5-*TCTV* using the IVIS imager, and additionally, imaging was evident in the secondary un-injected tumors (Figure 5B,D). No significant weight loss or overt signs of toxicity were observed in the treated mice as compared to the control mice (Figure S3). Additionally, we also checked the efficiency of our tripartite virus in a transgenic model of breast cancer (MMTV-PyMT model) with an intact immune system. The mice were monitored for tumor onset and tumors were either left untreated (control) or injected with Ad.5-*TCTV,* as described in “materials and methods,” once palpable tumors were observed. In order to evaluate both the tumor suppressive effects of MDA-7/IL-24 as well as the “bystander” anti-tumor effect of MDA-7/IL-24, at least 50% of the tumors formed were treated. We observed inhibition of tumor growth in all of the tumors including un-injected tumors in *TCTV* group compared to the controls (Figure 6A,B). Immunohistochemical findings show that MDA-7/IL-24 was expressed in the Ad.5-*TCTV*-injected tumors (Figure 6C). Additionally, to determine the imaging capacity of *TCTV* virus in MMTV-PyMT model a single mammary tumor on MMTV-PyMT mice was injected with the *TCTV* virus and the mouse was imaged the following day using bioluminescent imaging. The injected tumor was easily visualized within seconds. In order to determine whether other mammary tumors within the mouse could also be imaged, we masked the injected tumor (as described in methods) and imaged the mouse. We were able to visualize an adjacent uninjected tumor (Figure S4). In summary, these results suggest that *TCTV* retained its tumor-inhibiting activity in immune-compromised as well as immune-competent mice. Comparable anti-tumor activity was evident between *CTV*- and *TCTV*-treated groups confirming that the *TCTV* did not lose its primary anti-tumor activity or its “bystander” anti-tumor activity as a consequence of incorporating a third promoter element and imaging (luciferase) gene in this *TCTV* adenovirus. In addition, the *TCTV* was also able to selectively image tumors in vivo.

### 2.5. Ad.5-TCTV Induces Apoptosis and Inhibits Angiogenesis In Vivo

**Figure 7:**
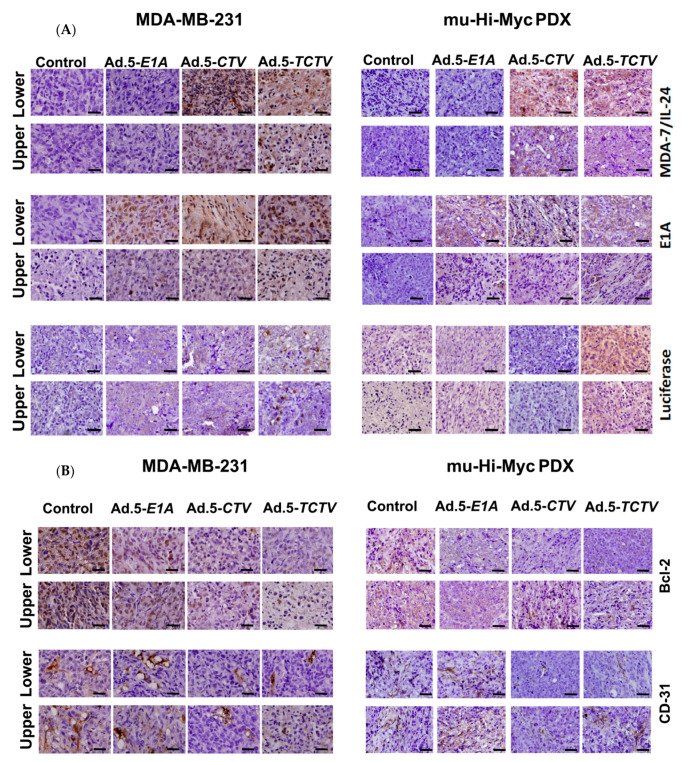
Ad.5-*TCTV* induces apoptosis and inhibits angiogenesis in vivo. MDA-MB 231 and mu-Hi-Myc PDX cancer cells were implanted subcutaneously in the lower and upper flanks of nude mice and the lower tumors were treated with eight intratumoral injections including solvent (mock treated control), Ad.5-*E1A* or Ad.5-*CTV* or Ad.5-*TCTV*. A total of five animals were studied in each group. Once the control animals’ tumors reached maximum allowable limit, tumors were collected, fixed in formalin, and embedded in paraffin. (**A**) Immunohistochemical (IHC) analysis of MDA-7/IL-24, adenovirus E1A, and Luciferase from tumor sections as indicated. (**B**) IHC analysis of Bcl-2 and CD-31 from tumor sections as indicated. Scale bar: 200 μM.

**Figure 8:**
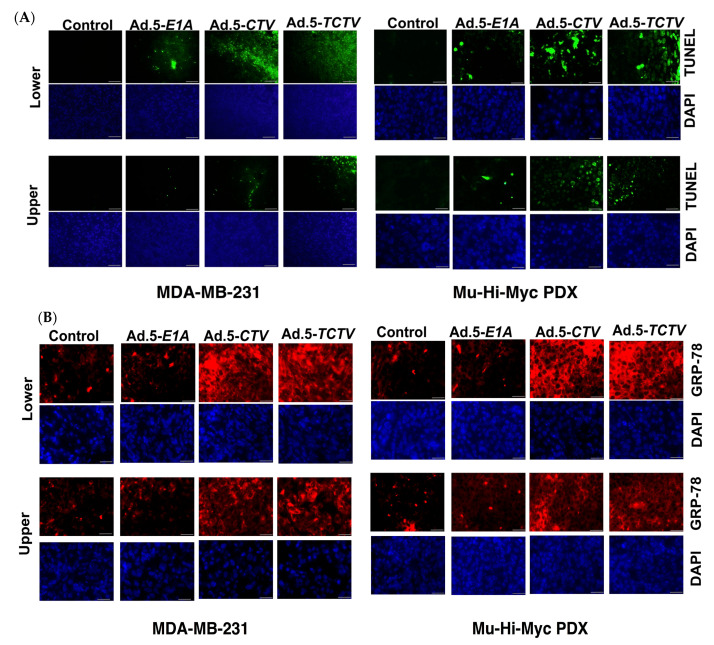
Ad.5-*CTV* and Ad.5-*TCTV* induces ER stress (GRP-78) and apoptosis (TUNEL) in breast and prostate tumors in nude mice. (**A**) MDA-MB 231 and mu-Hi-Myc PDX cancer cells were implanted subcutaneously in the lower and upper flanks of nude mice and lower tumors were infected with the indicated virus. A total of five animals were studied in each group. Once the control animals’ tumors reached maximum allowable limit, tumors were collected fixed in formalin and embedded in paraffin. TUNEL assays were performed according to the manufacturers protocol. Nuclei were stained with DAPI. Photographs were taken in ten random microscopic fields and representative pictures are shown. (**B**) Formalin and embedded in paraffin samples as prepared in **A** above, were stained for GRP-78 (an ER stress marker) and nuclei were stained with DAPI. Scale bar: 50 μM.

To explore further the potential mechanism of antitumor activity of Ad.5-*TCTV* and whether this effect involves induction of apoptosis in tumors and inhibited angiogenesis in vivo, tumor sections were evaluated using immunohistochemical assays (IHC) to determine the levels of MDA-7/IL-24, adenovirus E1A, Luciferase, CD-31, Bcl-2, and GRP-78 proteins (Figure 7A,B). Apoptotic content was also determined directly by the TUNEL assays. Consistent with our in vitro results, tumor sections from both directly treated tumors (lower flanks) as well as untreated tumors (upper flanks) in the Ad.5-*TCTV*-treated group showed increased staining for MDA-7/IL-24, adenovirus E1A, and Luciferase and the increase in MDA-7/IL-24 and adenovirus E1A was equivalent to that observed in tumor sections from Ad.5-*CTV*-infected tumors (Figure 7A). Also, the apoptotic index of tumor cells quantified by the number of TUNEL-positive cells increased similarly in both the Ad.5-*TCTV-* and Ad.5-*CTV*-infected tumor groups (Figure 8A). Consistent with previous reports showing that MDA-7/IL-24 inhibits angiogenesis [12,14,15], induces apoptosis by inhibiting Bcl-2 [27,66,67], and promotes ER stress [8,25,36,51,52,53,54], Ad.5-*TCTV* infection reduced CD-31 and Bcl-2 levels and increased GRP-78 protein levels (Figure 8B) in treated tumors. Additionally, these anticancer activities were also evident in the untreated upper flank tumors in the lower flank-treated groups infected with Ad.5-*CTV* and Ad.5-*TCTV* (Figure 7B and Figure 8). These observations demonstrate that integrating an imaging component in the bipartite *CTV* does not compromise the anticancer and tumor anti-angiogenesis activity of *TCTV*.

## 3. Discussion

Although potentially an exemplary way to treat cancer, therapeutic applications of cancer-selective replication-incompetent viruses delivering therapeutic gene products as well as conditionally replication-competent viruses to destroy tumor cells have not performed up to expectations in clinical trials in patients with diverse cancers [68,69,70]. When combining these engineered viruses with additional therapeutic agents (including radiation, chemotherapy, immunotherapy, and gene therapy), in specific instances enhanced clinical endpoints have been achieved, but even these combinations have not proven universally effective, particularly in the context of patients with metastases [68,71]. A further limitation of the current therapies for cancer metastases, which is the primary cause of morbidity (estimated to be 90%) in patients with solid cancers, is the inability to accurately locate metastatic tumor cell populations and to appropriately deliver therapeutic payloads in tumor cells or the tumor microenvironment [2,3,4]. 

Various serotypes of adenoviruses have received significant attention as vectors for inducing cancer-cell lysis and delivery of therapeutic genes [25,44,48,58,59,60,64,65,68,71]. These viruses are easy to genetically modify and manufacture [68,69,70]. In early therapeutic trials, most of these adenoviruses were replication-incompetent, but currently the majority of therapeutic applications exploit promoters that can drive adenovirus E1A and E1B gene expression in a cancer-specific manner resulting in conditional replication [60,68,69,70,71]. Considering the limitations of the current therapeutic viruses in cancer therapy, our research program focuses on developing strategies to enhance viral infectivity, target systemic delivery of viruses (in a manner preventing trapping in the liver or clearance by the immune system), and developing complex viruses that have multiple therapeutic components and attributes (including cancer-selective replication, delivery of a therapeutic cytokine with broad “bystander” antitumor activity, and incorporation of an imaging agent- “theranostic” viruses). 

We previously described a cancer therapeutic bipartite adenovirus that selectively replicates in tumor cells and simultaneously produces a ubiquitous cancer suppressor gene *mda-7/IL-24*, a cancer terminator virus (*CTV*) [44,45,58,59,60,64,65,71]. This virus has shown therapeutic activity toward a broad-spectrum of cancers regardless of anatomic origin and does not harm the normal tissue, effectively promoting tumor growth suppression in preclinical animal models [44,48,58,59,60,64,65]. The *CTV* produces selective cancer destruction through cytolysis (promoted by viral replication) and by the multiple mechanisms in which *mda-7/IL-24* can eliminate cancer cells. These include: Selective direct killing of cancer cells of diverse origin by inducing apoptosis and/or toxic autophagy; destroying localized tumors and distant metastatic tumor cells through “bystander” activity as a secreted cytokine; promoting tumor cell killing through immune-based mechanisms as a potent immune-modulating agent; suppressing tumor growth by inhibiting tumor angiogenesis; and synergizing with radiation, chemotherapy, immunotherapy, and antibody-based therapies that promote cancer cell death [7,8,13,15,56,72]. These properties make the *CTV* a potentially useful reagent for viral-based therapy of cancer [58,59,60,71]. 

The focus of the current work was to improve further the *CTV* by embodying it with an additional therapeutic property, the capacity to selectively image cancer in vivo, i.e., converting the *CTV* into a tripartite “threranostic” adenovirus (*TCTV*). The key functional component of a *CTV,* as well as a *TCTV,* involves genetic sequences that control cancer-specific/cancer-selective gene expression. To achieve this objective, we relied on the minimal *PEG*-3 promoter [62,63], which is regulated by AP1 and PEA3 transcription factors, and expresses selectively in both cancerous murine and human cells, with minimal expression in normal cells [44,63,64,65]. Additionally, we have shown that the minimal *PEG*-3 promoter, an essential part of the *CTV*, can also be used to image metastatic cancer cells (including prostate, breast, and melanoma) non-invasively (using *luciferase* or *thymidine kinase* reporter genes) in pre-clinical animal models [1,2,3]. Based on these considerations, we initially attempted to generate a tripartite adenovirus in which both the viral Ad.5 E1A gene-regulating replication and the imaging *luciferase* gene were regulated independently by two copies of the minimal *PEG*-Prom, and also containing *mda-7/IL-24* transcriptionally regulated by the cytomegalovirus (*CMV*) promoter. This approach was not successful in generating constructs that could generate complete functional adenoviruses, most likely because of homologous recombination between the *PEG*-3 promoter sequences linked independently to the Ad.5 E1A gene and the *luciferase* gene. Accordingly, we devised a strategy that employed two different cancer-selective promoters to independently drive Ad.5 E1A and *luciferase*. For this purpose, we retained the minimal *PEG*-3 promoter to drive the Ad.5 E1A gene and chose a truncated *tCCN1*-promoter that displays elevated expression in cancer cells [64], to drive *luciferase* gene expression, while again retaining the CMV-driven *mda-7/IL-24* gene, i.e., an Ad.5-*TCTV* (Figure 1). 

Expression of appropriate genes by the *TCTV* and selective biological effects on cancer cells in vitro and in vivo were evaluated in breast and prostate cancer and normal epithelial cells. Infection of cancer cells with Ad.5-*TCTV* or Ad.5-*CTV* produced comparable amounts of the Ad.5 E1A and *mda-7/IL-24* transgenes, as well as changes in key apoptosis-related molecules (Figure 1 and Figure 3). Moreover, both viruses displayed similar in vitro anti-tumor growth inhibitory effects, without suppressing growth of normal breast or prostate epithelial cells (Figure 2). An important therapeutic property of the *CTV* is the ability to induce potent “bystander” antitumor effects in vivo in pre-clinical animal models [15,17,52,53,55,65], which was evaluated using Ad.5-*TCTV* (Figure 5 and Figure 6). Bystander activity was confirmed after establishing human breast and murine-PDX tumors on the opposite upper and lower flanks of nude mice followed by treatment of only lower flank tumors with Ad.5-*TCTV* or Ad.5-*CTV* (Figure 5). This resulted in apoptosis induction (monitored by TUNEL assay) and a direct anti-tumor effect that was evident in both the lower (injected) and upper (non-injected) tumors (Figure 3 and Figure 8A). Repeated injection of a subset of spontaneously developing mammary tumors in MMTV-PyMT transgenic mice with the Ad.5-*TCTV* resulted in tumor growth suppression in both injected and non-injected mammary tumors (Figure 6). Uniquely, infection with the *TCTV* was visible by bioluminescence imaging in both the lower injected and upper un-injected tumors in nude mice, and adjacent tumors in a syngeneic model (MMTV-PyMT) indicating that the luciferase component of the *TCTV* was also functional (Figure 5 and Figure S4). These findings confirm the successful engineering of a functional tripartite “theranostic” adenovirus.

Additional pieces in the puzzle to more effectively use oncolytic adenoviruses therapeutically, which affects the utility of the Ad.5-*TCTV* and the Ad.5-*CTV*, involves overcoming problems with efficient infectivity and systemic delivery to tumor cells, i.e., avoiding trapping in the liver and clearance of viruses by the immune system [60,71,73]. Variable degrees of success have been achieved in enhancing infectivity and promoting systemic delivery of adenoviruses by modifying the structure of the capsid, and creating chimeric viruses such as Ad.5/Ad.3 or Ad.5/Ad.48 viruses [25,48,60,73,74,75] and the use of physical and chemical modifications of Ad particles [76]. Additional strategies for systemic virus delivery employ biologically derived extracellular vesicles [77], nanoparticles [78], and microbubbles combined with ultrasound (the ultrasound-targeted microbubble-destruction (UTMD) approach) [25,60,64,71]. Two strategies that could help expand the use of the Ad.5-*TCTV,* as well as the Ad.5-*CTV,* preventing trapping in the liver and clearance by the immune system that deserve further consideration are nanoparticles and the UTMD approach. Nanoparticles have been found to be a powerful method to deliver therapeutic payloads to tumor sites, including targeting viruses [78,79,80]. This method of delivery could enhance the effectiveness of our theranostic tripartite cancer terminator virus by delivering it to difficult-to-treat regions in the body. In our laboratory, we have pioneered the use of UTMD to deliver therapeutic viruses systemically to maximize the targeting of the virus near the tumor and its vasculature limiting immune clearance [25,60,64,71]. We demonstrated previously that virus conjugated with microbubbles could be delivered through tail vein injection to the prostate [25]. After intravenous injections the microbubbles with their payload circulate in the bloodstream and ultrasound is used to disrupt the microbubbles to release the virus where intended, e.g., in the prostate region [25,60,64,71]. Based on these studies, the *TCTV* could be delivered to tumors and their microvasculature and visualized, while displaying comparable anticancer activity as demonstrated earlier with the *CTV* [44,58,59,60,65,71].

## 4. Material and Methods

### 4.1. Cells and Reagents

Breast cancer, MDA-MB-231 and SUM-159, and prostate cancer, DU-145 and PC-3, cell lines were obtained from ATCC. RWPE-1 normal immortal human prostate epithelial cells and normal early passage primary human mammary epithelial cells (HMEC) were also obtained from ATCC. A murine PDX cell line was established from Hi-Myc prostate cancer, mu-Hi-Myc PDX. The collective culture length of the cancer, mu-Hi-Myc PDX and immortal cells was less than 6 months once revived from liquid nitrogen storage. Normal early passage primary mammary cells were grown in specific media recommended by the manufacturer for four passages following establishment. Early passaged cells were used for all experiments and they were regularly tested for mycoplasma contamination. Suggested culture media supplemented with 10% FBS, 50 units/mL penicillin, and 50 μg/mL streptomycin (Life Technologies Inc., Carlsbad, CA, USA) were used for cell cultures. Cells were incubated in a humidified incubator with 5% CO_2_ at 37 °C. The antibodies specific for: Bcl2, PARP, Luciferase (Cell signaling technology, Danvers, MA, USA); adenovirus E1A (Miilipore, Burlington, MA, USA); GADD153, GRP-78 (Santa Cruz Biotechnology, Dallas, TX, USA); MDA-7/IL-24 (#K101; GenHunter, Nashville, TN, USA); horseradish peroxidase (HRP)-conjugated secondary antibodies (Dako, Santa Clara, CA, USA); and β-actin (#NB600-501; Novus Biologicals, Inc., Littleton, CO, USA) were used in this study. First Strand cDNA Synthesis Kit (Applied Biosystems, Foster City, CA, USA), In Situ Cell Death Detection Kit, Fluorescein (#11684795910; Roche Applied Science, St. Louis, MO, USA), and MTT cell growth assay Kit (#CT02; Millipore Corporation, Burlington, MA, USA) were the other reagents used in this study.

### 4.2. Engineering Ad.5-TCTV

The schematic sequence of the *TCTV* is shown in Figure 1A. The genome of Ad.5-*PEG-E1A-tCCN1-Luc-CMV-mda-7/IL-24* was generated in consecutive steps as depicted in Figure 1B. pAd.5-E3-luc-*mda*-7 genome was constructed by homologous recombination of pAd.5 genome plasmid with a shuttle vector (pShuttlE3) having both the *mda-7/IL-24* expression cassette as well as the tCCN1-Luc expression cassette. The resulted pAd.5-E3-luc-*mda*-7 plasmid was recombined with another shuttle vector (pShuttlE1 plasmid) with a minimal PEG-3 promoter controlling E1A and E1B genes resulting in Ad.5-*PEG-E1-luc-mda-7* (Ad.5-*TCTV*) genome plasmid. The plasmid was digested with *Pac*I to release viral ITRs and transfected into A549 cells to rescue the conditionally replication competent adenovirus, Ad.5-*TCTV* (Figure 1B).

### 4.3. Cell Proliferation Assay (MTT Assay)

An adjusted 3-(4,5-dimethylthiazol-2-yl)-2,5-diphenyltetrazolium bromide (MTT) assay was used to determine the cell proliferation as described previously [22,36]. Briefly, 2000 cells per well were plated in 96-well plates and treated with Ad.5-*Vec*, Ad.5-*E1A*, or the indicated doses of Ad.5-*CTV* or Ad.5-*TCTV* and incubated for 72 h. MTT reagent was then added, and cells were incubated for an additional 4 h. Formazan crystals were dissolved using DMSO and absorbance was read at 550 nm using a microplate spectrophotometer. Results are an average from 8-wells each and expressed graphically.

### 4.4. Terminal Deoxy Nucleotidyl Transferase-Mediated Nick-End Labeling (TUNEL) Assay

Apoptosis induction in cancer cells or tumor sections of mock-treated or infected with Ad.5-*Vec,* Ad.5-*E1A*, Ad.5-*CTV,* or Ad.5-*TCTV* was assessed with terminal deoxynucleotidyl transferase-mediated dUTP nick end labeling (TUNEL) enzyme reagent. The experiment was performed as described earlier [66] following the manufacturer’s instructions. Briefly, 5 × 10^3^ cancer cells were plated in 4-well chamber slides and mock-treated or infected with Ad.5-*Vec*, Ad.5-*E1A*, Ad.5-*CTV,* or Ad.5-*TCTV* for 72 h. These cells were then fixed at room temperature for 1 h in 4% buffered paraformaldehyde (tissue sections were rehydrated by standard procedure) and permeabilized with 0.1% Triton-X 100 in 0.1% sodium citrate in PBS for 2 min (for cells) or 10 min (for tissue sections) on ice. TUNEL reaction mixture was added to these permeabilized samples and were incubated for 1 h in the dark in a humidified atmosphere at 37 °C. These slides were counterstained with DAPI and images were captured with an Olympus research fluorescence microscope and a representative image is shown. 

### 4.5. Western Blotting

Western blotting analysis was performed as described previously [16,22,26]. RIPA (radio-immunoprecipitation assay) lysis buffer containing phosphatase and protease inhibitors was used to lyse the mock-treated and Ad.5-*Vec-*, Ad.5-*E1A-*, Ad.5-*CTV-,* or Ad.5-*TCTV*-infected cells. Total protein was measured using BCA reagent and equal total protein was resolved by SDS-PAGE and transferred to a polyvinylidene difluoride (PVDF) membrane. The membrane was then blocked with 5% non-fat dry milk or 5% BSA for 1 h. Blocked membranes were incubated overnight with primary antibodies followed by HRP-conjugated secondary antibodies. An ECL reagent was used to detect chemiluminescent signals and captured using X-Ray films. Equal loading was confirmed by reprobing all the blots with β-actin antibody.

### 4.6. In Vivo Studies

All in vivo experiments using mice were approved by the Institutional Animal Care and Use Committee (IACUC) of Virginia Commonwealth University, Richmond, VA, USA, protocol number: AM10183. A subcutaneous tumor model was used to directly evaluate the effect of Mock-treated or infection with Ad.5-*Vec*, Ad.5-*E1A*, Ad.5-*CTV,* or Ad.5-*TCTV* on tumor growth in vivo. About 2 × 10^6^ breast/prostate cancer cells were subcutaneously implanted on the upper and lower flanks of 4- to 6-week-old athymic nude mice. When the tumor reached palpable size around seven-days after tumor cell implantations, the tumors that developed on the lower flank were treated with eight intratumoral injections of Ad.5-*Vec*, Ad.5-*E1A*, Ad.5-*CTV,* or Ad.5-*TCTV* for 3 weeks (3 injections for 2 weeks and 2 injections in the last week). Tumor development was monitored every alternate day until the end of the experiment and measured using calipers. We used two sets of mice for each group, 2 days after the final treatment one set of mice were euthanized (1 mouse from each group), and another set (*N* = 5) was followed until the control tumor group reached the IACUC established end point where it needed to be sacrificed. Tumors treated with Ad.5-*TCTV* were also followed using IVIS imaging. For BLI imaging, D-Luciferin, the substrate of luciferase, was injected intraperitoneally into mice at a dose of 150 mg/kg. After 10 min the mice were anesthetized and placed on the imaging stage of the IVIS apparatus in the prone (subcutaneous model) or the supine position (MMTV-PyMT model). Images were collected by setting exposure time as constant (1 min) using the IVIS Imaging System (Xenogen, Alameda, CA, USA). After completion of the experiment, the tumors were fixed and the sections were used for immunohistochemical (IHC) analysis.

MMTV-PyMT (mouse mammary tumor virus polyoma middle T) mouse model was used to evaluate the therapeutic efficacy of the Ad.5-*TCTV*. These transgenic mice develop mammary tumors spontaneously in all the mammary glands within 2–3 months of age. Once a palpable tumor was observed in any mammary gland in PyMT mice, Ad.5-*TCTV* (1 × 10^8^ VP in 50 µL) was injected intratumorally to determine the potential tumor suppressive effects of MDA-7/IL-24 and these mice were followed as described previously [33]. 50% of the tumors that formed were injected with Ad.5-*TCTV* (in mice having 1 or 2 tumors, 1 tumor was injected; in mice having 3 or 4 tumors, 2 tumors were injected and so on) as the mice developed tumors in the mammary glands. The other 50% of tumors within a particular mouse were left untreated to determine the bystander anti-tumor properties of MDA-7/IL-24. The same tumors within a single mouse were injected repeatedly by maintaining a log for the tumors. Each treated tumor in a mouse received a maximum of ten injections (depending on when palpable tumors were observed) over a 4-week period. When the tumors in control mice reached the IACUC end point, mice were euthanized, tumors were collected, formalin-fixed, paraffin-embedded and sectioned, and immunohistochemistry was performed following the standard procedures. 

To determine the potential imaging capabilities of luciferase a single mammary tumor on MMTV-PyMT mice was injected with Ad.5-*TCTV* and the mouse was imaged the following day using BLI. In order to determine whether other mammary tumors within the mouse could also be imaged, we masked the injected tumor (because it showed high luminescence) and imaged the mouse. The tumors were harvested and re-visualized using BLI. 

### 4.7. Immunohistochemical Analysis 

When the tumors in control mice reached maximum allowable size, mice were euthanized and tumors were collected, fixed in formalin, and embedded in paraffin. The sections were deparafinized, rehydrated, and were permeabilized with Citrate/Triton-X buffer (0.1% Triton X-100 in PBS) for 30 min. These sections were then blocked with 2% goat serum and 1% bovine serum albumin in PBS for 1 h at room temperature and incubated with indicated antibodies [anti-E1A (1:100), anti-MDA-7/IL-24 (1:100), anti-Bcl-2 (1:200), anti CD-31 (1:100), or anti-luciferase (1:200)], overnight at 4 °C, these slides were then washed with PBS and incubated with suitable secondary antibodies for an additional one hour. Once the incubation was completed, the sections were treated with 3,3′-diaminobenzidine (DAB) solution as chromogen and nuclei were counterstained with hematoxylin and dehydrated as per standard protocol. Sections were mounted with mounting solution and analyzed using an inverted microscope.

### 4.8. Statistical Analysis

At least three independent experiments were performed in triplicate and the data were presented as mean ± S.D. Statistical analysis was performed for multiple comparisons using one-way ANOVA combined with the Tukey post-hoc test. Statistical differences are displayed at probability levels of *p* < 0.05, *p* < 0.01, and *p* < 0.001.

## 5. Conclusions

In summary, the “theranostic” tripartite cancer terminator virus have many benefits over conventional therapies as it can use diverse mechanisms to target and eradicate cancers. Using a single vector, we were able to detect the injected and adjacent tumors in both immunocompromised and immunocompetent settings, deliver therapeutics and monitor therapy responses. The capability of these viruses to non-invasively image cancers in pre-clinical models and throughout clinical testing will increase the use and development of such types of viruses. Furthermore, sequential imaging of a tumor will be beneficial to evaluate the response of therapeutic agents non-invasively. As there are some limitations in using luciferase as a reporter gene in the clinical setting, we further plan to develop oncolytic viruses capable of imaging and treating cancer at the same time using a clinically relevant imaging modality like HSV-TK and SPECT-CT. Additionally one can build next-generation tripartite oncolytic viruses by enhancing cancer specificity and robustness of promoter activity. With future iterations of the *TCTV,* including structural enhancement of the adenovirus to increase infectivity of cancer cells and incorporation of a reporter gene encoding a product that can be safely used in patients [4], new tripartite theranostic viruses could provide powerful weapons for both the diagnosis and therapy of local and metastatic cancers.

## Data Availability

The data presented in this study are available on request from the corresponding author.

## Supplementary Materials

The following are available online at https://www.mdpi.com/2072-6694/13/4/857/s1. Figure S1: (A) Densitometry analysis of Western blots in Figure 1D left panel, (B) right panel. Figure S2: (A) Densitometry analysis of Western blots in Figure 3A left panel, (B) Densitometry analysis of Western blots in fig 3A right panel, (C) Densitometry analysis of Western blots in fig 3C left panel, (D) Densitometry analysis of Western blots in Figure 3C right panel. Figure S3: Ad.5-E1A, Ad.5-CTV or Ad.5-TCTV did not cause any significant alterations in the body weight of treated animals as compared to control group. Figure S4: Injection of a single tumor can light up an adjacent tumor within the same mouse in MMTV-PyMT model. (A) Representative bioluminescent images showing both injected and uninjected tumors being visualized using bioluminescent imaging. (B) Representative bioluminescent images of the dissected tumors in vitro.
