# Supplementary material for: Theranostic Tripartite Cancer Terminator Virus for Cancer Therapy and Imaging
Source: Cancers (Basel). 2021 Feb 18;13(4):857. doi: 10.3390/cancers13040857 (PMC7922065; doi:10.3390/cancers13040857)
Supplement: Supplementary file 1 [file cancers-13-00857-s001.pdf]

cancers

Supplementary materials

Theranostic Tripartite Cancer Terminator Virus for Cancer Therapy and Imaging

Praveen Bhoopathi [1,†,*], Anjan K. Pradhan [1,†], Santanu Maji [1], Swadesh K. Das [1,2,3], Luni Emdad [1,2,3] and Paul B. Fisher [1,2,3,*]

[1] Department of Human and Molecular Genetics, Virginia Commonwealth University, School of Medicine, Richmond, VA 23298, USA; anjan.pradhan@vcuhealth.org (A.K.P.); santanu.maji@vcuhealth.org (S.M.); swadesh.das@vcuhealth.org (S.K.D.); luni.emdad@vcuhealth.org (L.E.)
[2] VCU Institute of Molecular Medicine, Virginia Commonwealth University, School of Medicine, Richmond, VA 23298, USA
[3] VCU Massey Cancer Center, Virginia Commonwealth University, School of Medicine, Richmond, VA 23298, USA
* Correspondence: paul.fisher@vcuhealth.org (P.B.F.); praveen.bhoopathi@vcuhealth.org (P.B.)
† Contributed equally.

Supplementary materials

Citation: Bhoopathi, P.; Pradhan, A.K.; Maji, S; Das, S.K.; Emdad, L.; Fisher, P.B. Theranostic Tripartite Cancer Terminator Virus for Cancer Therapy and Imaging. *Cancers* **2021**, *13*, x. https://doi.org/10.3390/xxxxx

Academic Editor:

Received: 24 December 2020
Accepted: 15 February 2021
Published: date

Publisher's Note: MDPI stays neutral with regard to jurisdictional claims in published maps and institutional affiliations.

Copyright: © 2020 by the authors. Submitted for possible open access publication under the terms and conditions of the Creative Commons Attribution (CC BY) license (http://creativecommons.org/licenses/by/4.0/).

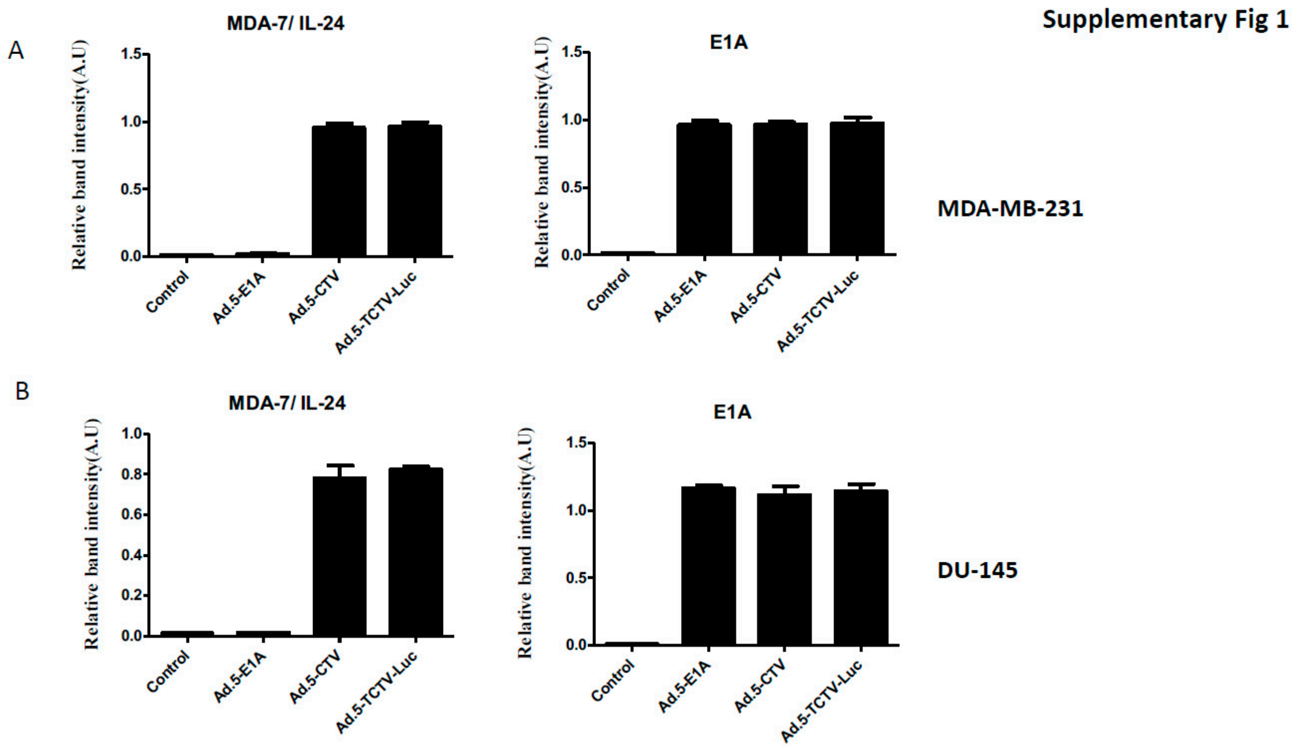

Figure S1. (**A**) Densitometry analysis of Western blots in figure 1D left panel, (**B**) right panel.

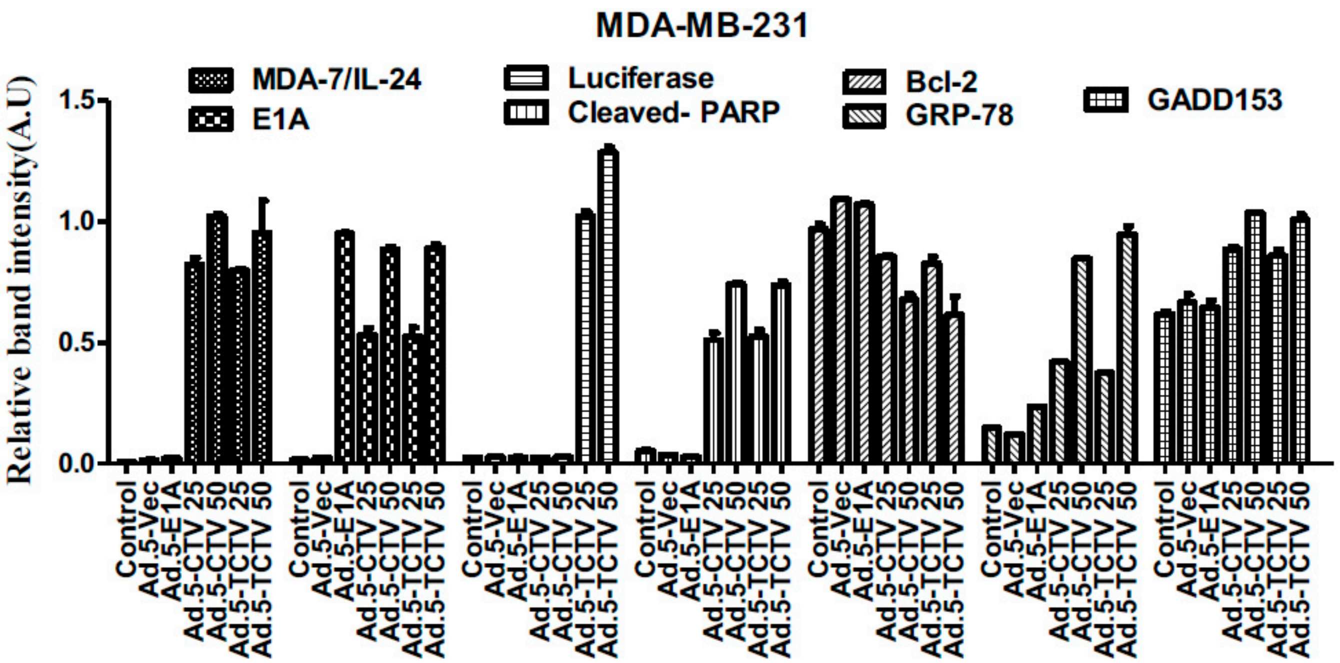

(**A**)

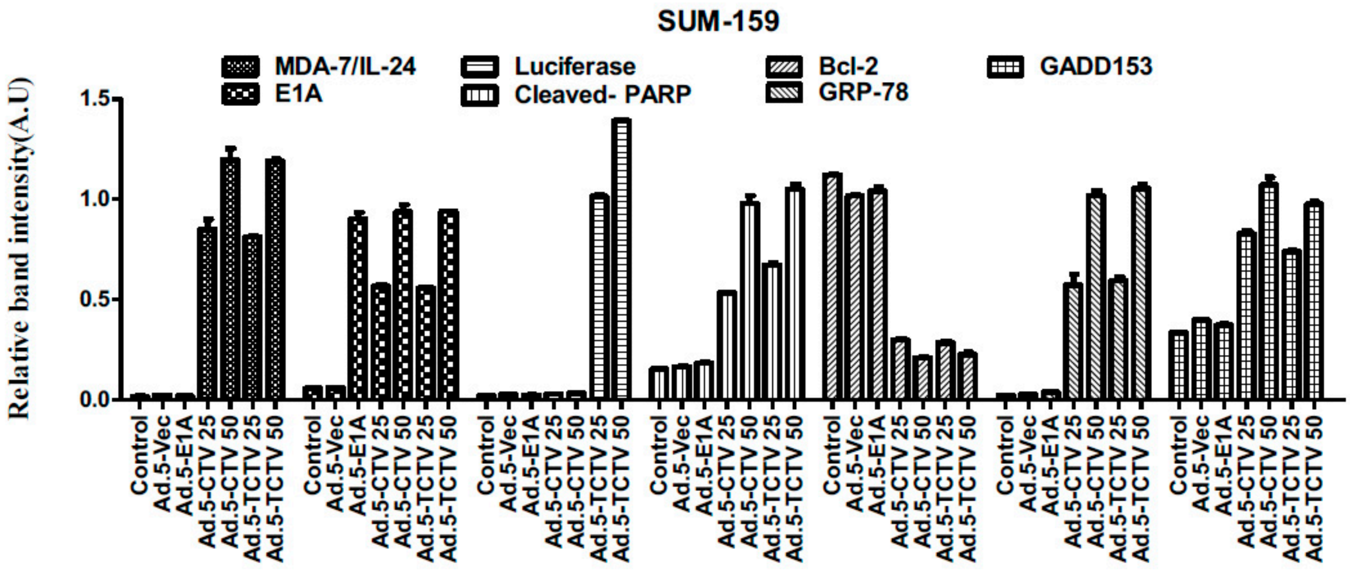
SUM-159
MDA-7/IL-24
E1A
Luciferase
Cleaved- PARP
Bcl-2
GRP-78
GADD153
Relative band intensity(A.U)
1.5
1.0
0.5
0.0
Control
Ad.5-Vec
Ad.5-E1A
Ad.5-CTV 25
Ad.5-CTV 50
Ad.5-TCTV 25
Ad.5-TCTV 50

(**B**)

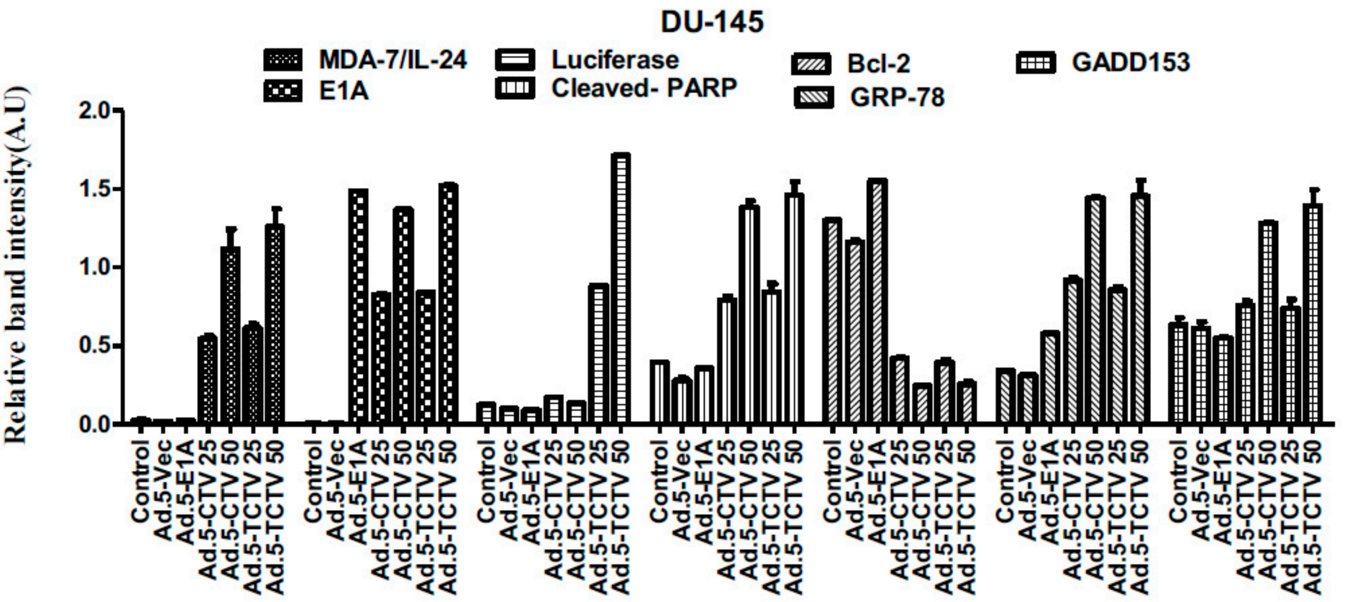
DU-145
MDA-7/IL-24
E1A
Luciferase
Cleaved- PARP
Bcl-2
GRP-78
GADD153
Relative band intensity(A.U)
2.0
1.5
1.0
0.5
0.0
Control
Ad.5-Vec
Ad.5-E1A
Ad.5-CTV 25
Ad.5-CTV 50
Ad.5-TCTV 25
Ad.5-TCTV 50

(**C**)

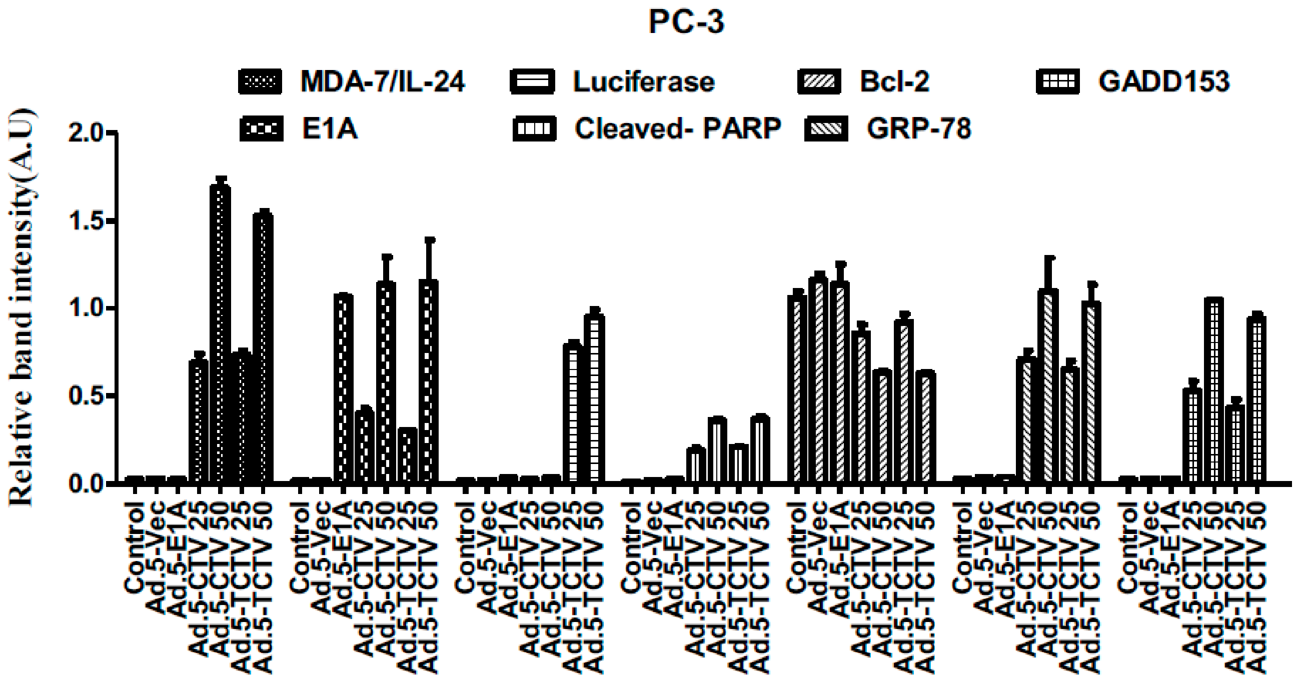

(**D**)

Figure S2. (**A**) Densitometry analysis of Western blots in figure 3A left panel, (**B**) Densitometry analysis of Western blots in fig 3A right panel, (**C**) Densitometry analysis of Western blots in fig 3C left panel, (**D**) Densitometry analysis of Western blots in figure 3C right panel.

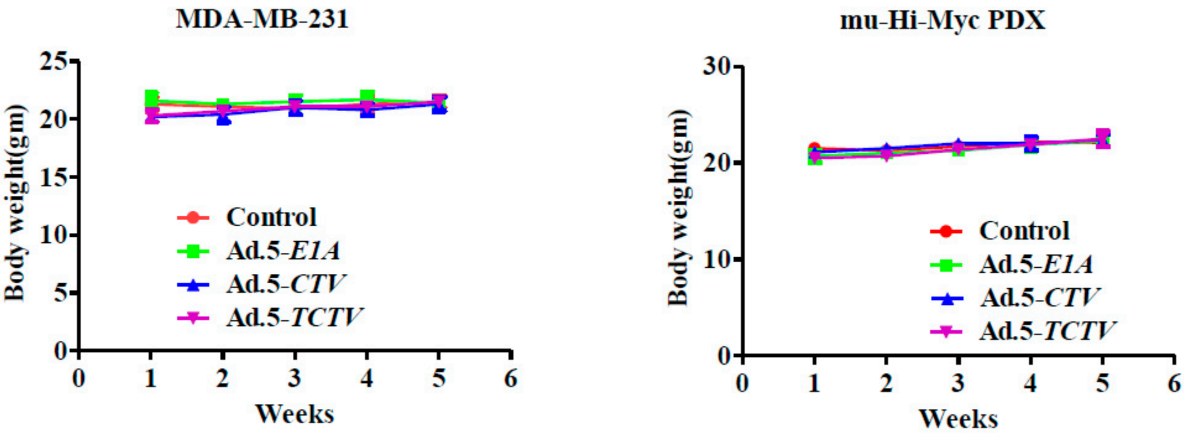

Figure S3. Ad.5-*E1A*, Ad.5-*CTV* or Ad.5-*TCTV* did not cause any significant alterations in the body weight of treated animals as compared to control group.

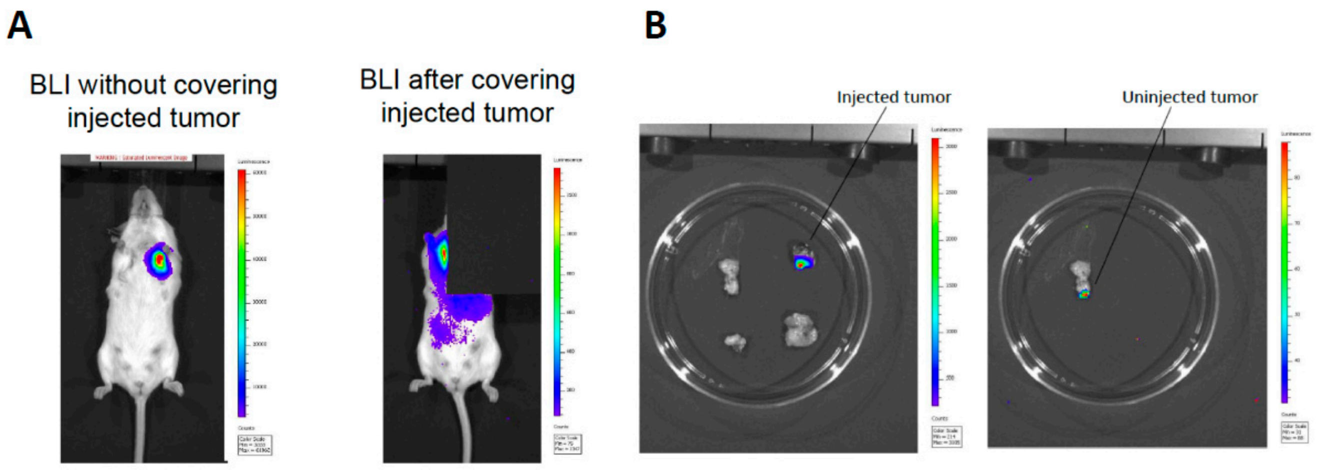

Figure S4. Injection of a single tumor can light up an adjacent tumor within the same mouse in MMTV-PyMT model. (**A**) Representative bioluminescent images showing both injected and.uninjected tumors being visualized using bioluminescent imaging. (**B**) Representative bioluminescent images of the dissected tumors *in vitro*.

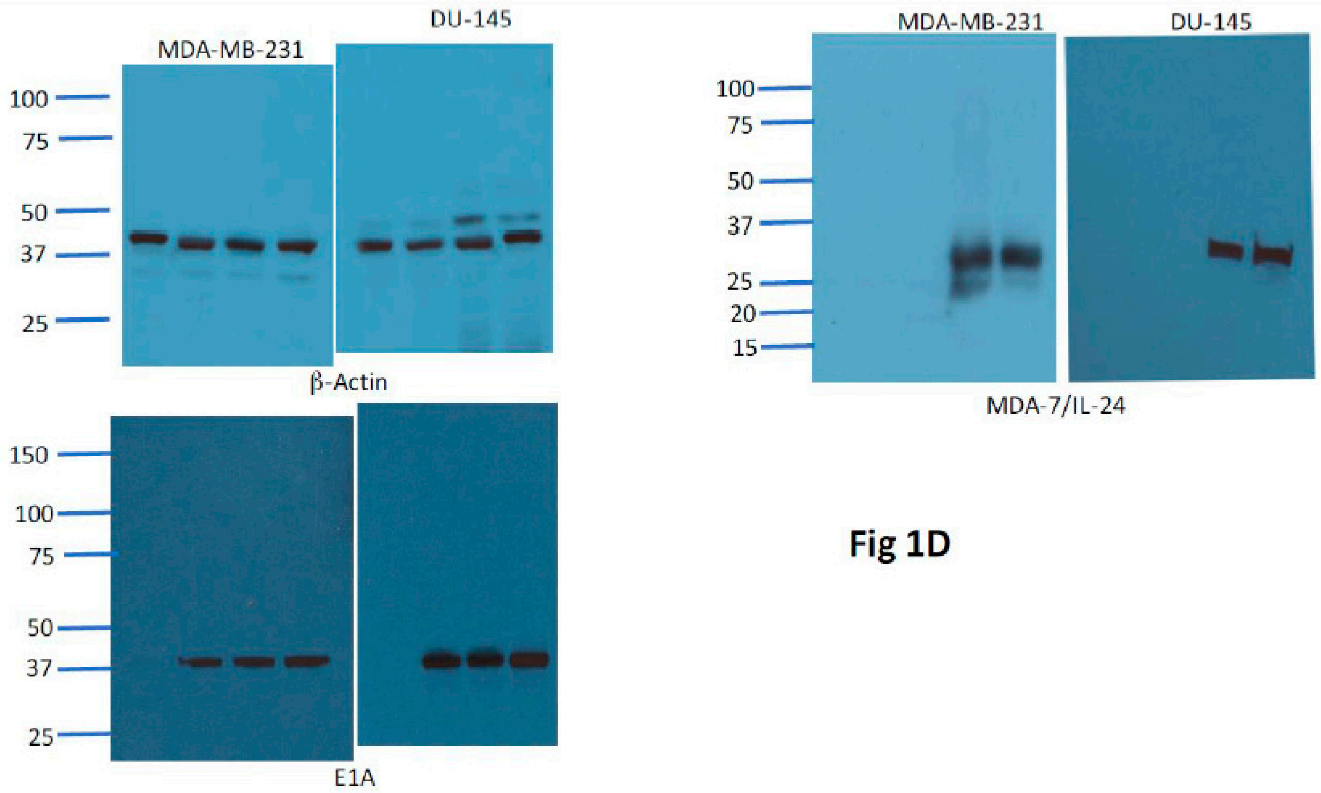

Fig 1D

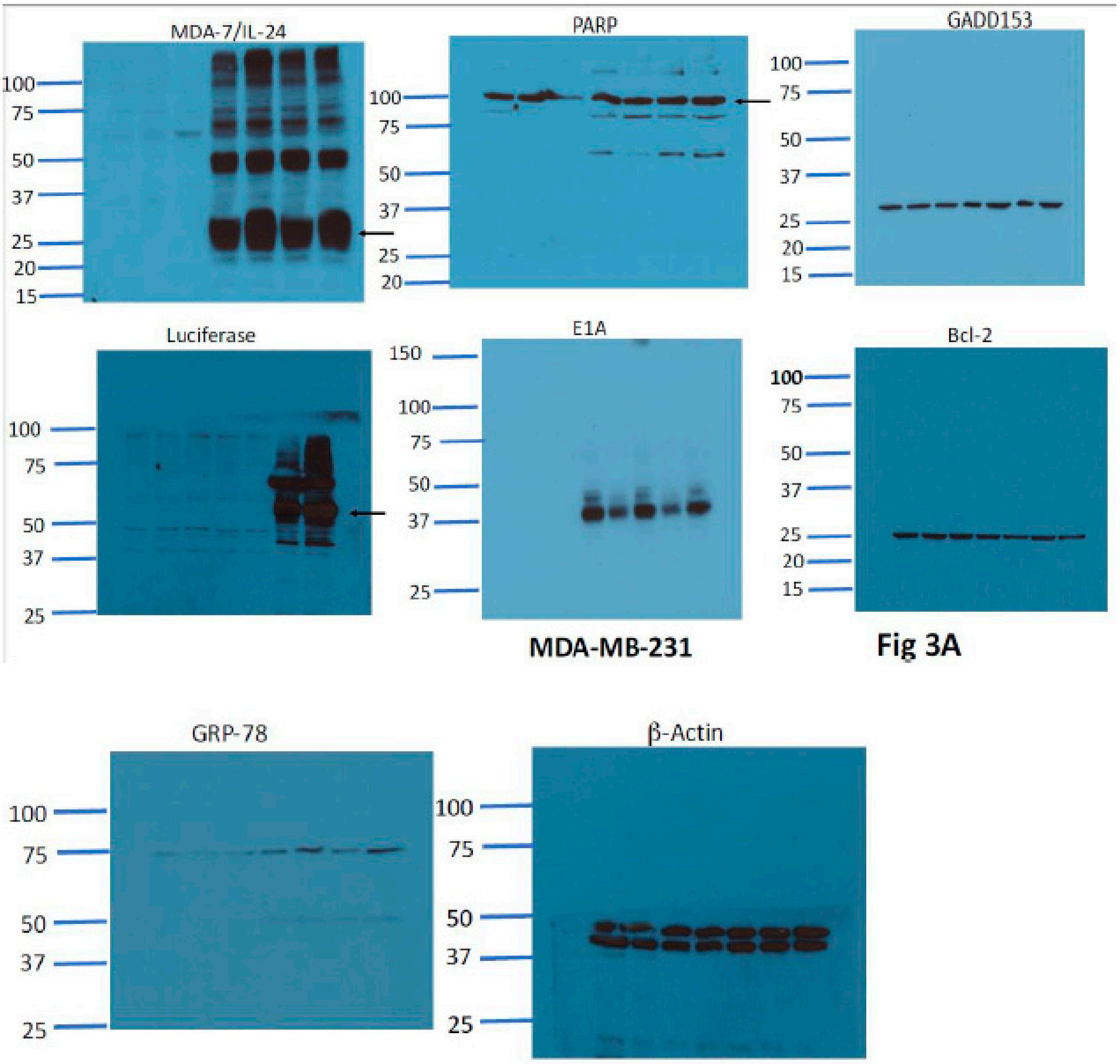

MDA-MB-231

Fig 3A

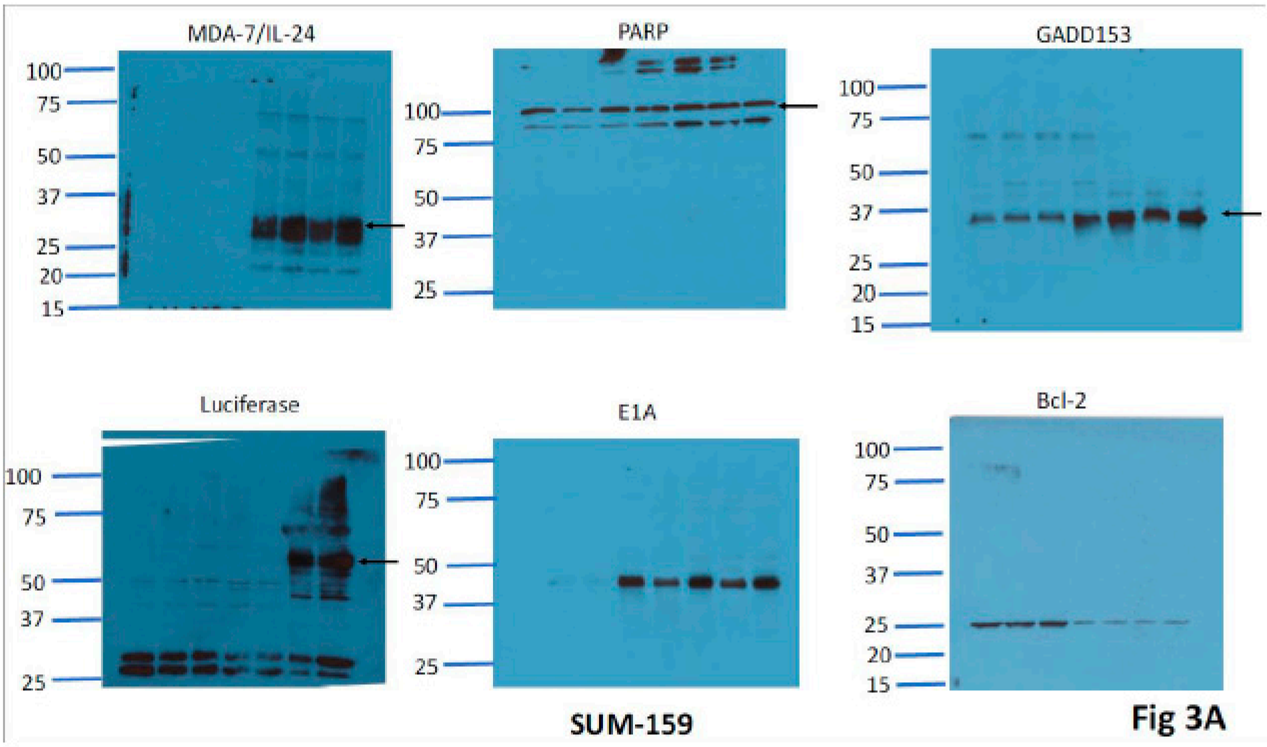

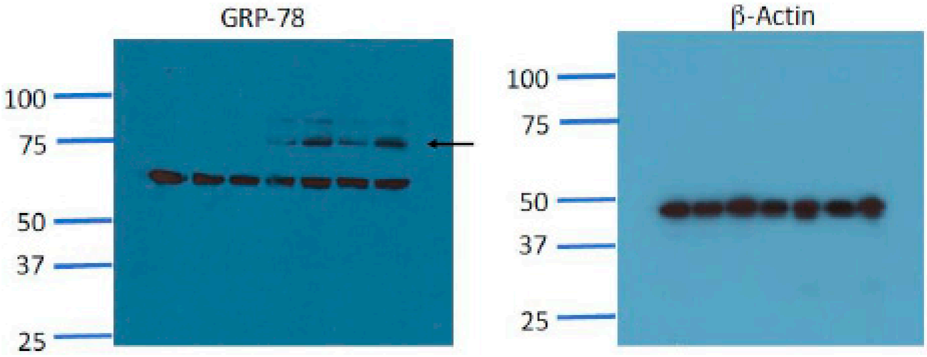

SUM-159

Fig 3A

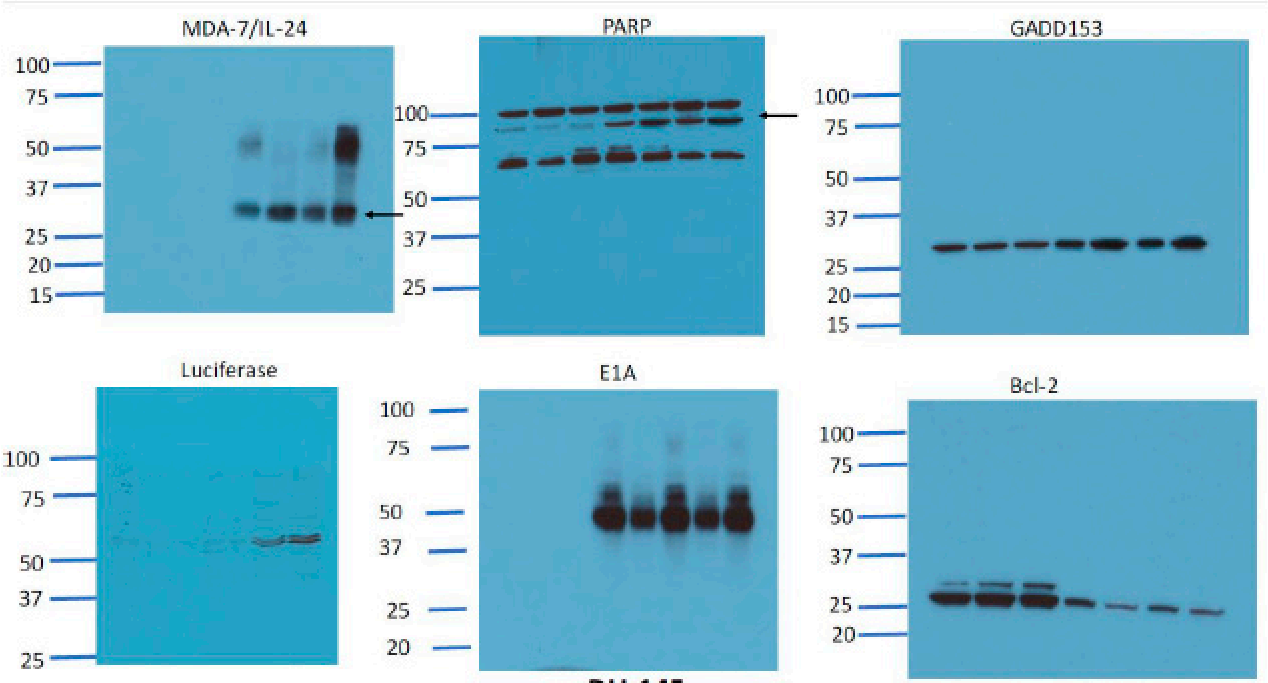

DU-145

Fig 3C

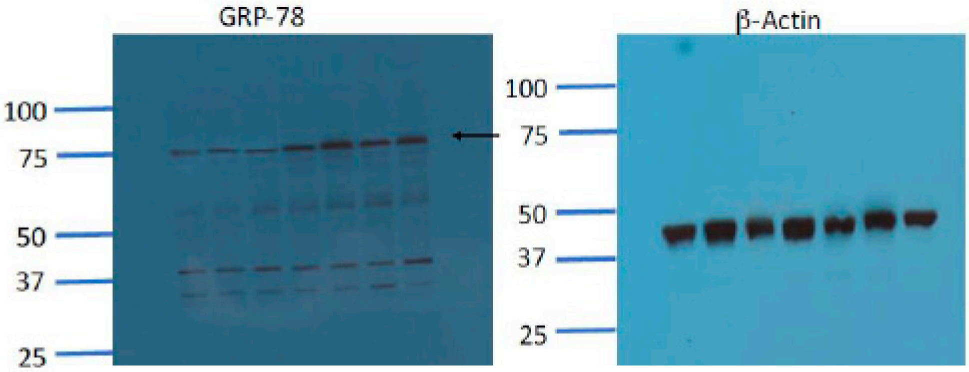

DU-145

Fig 3A

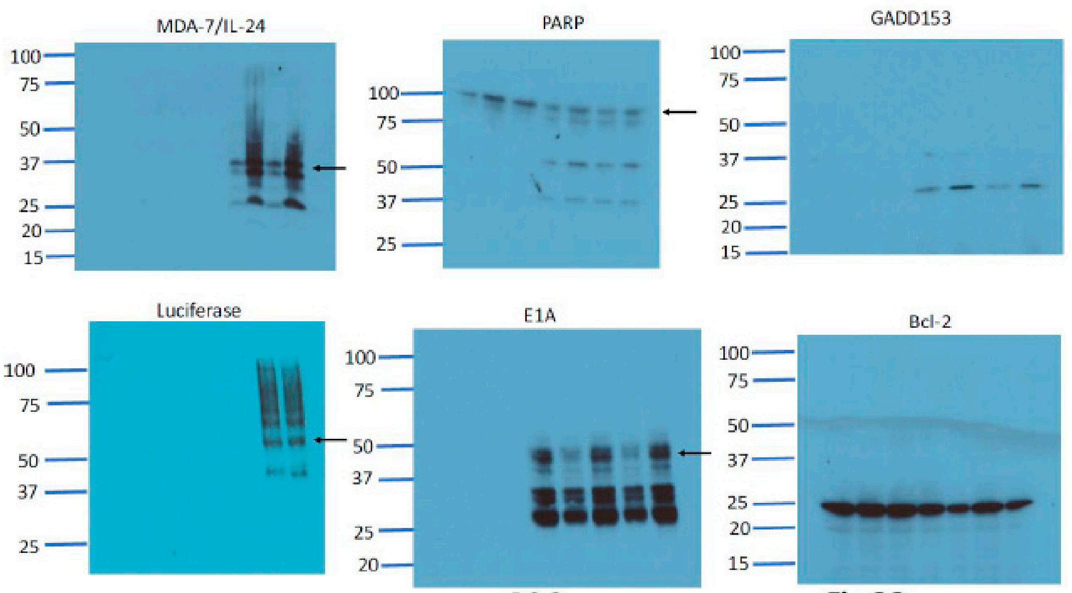

PC-3

Fig 3C

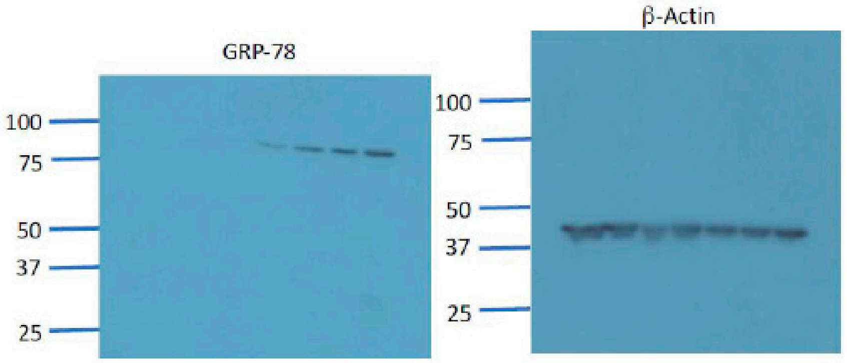

PC-3

Fig 3C
